# Effects of *In vivo* Emergent Tigecycline Resistance on the Pathogenic Potential of *Acinetobacter baumannii*

**DOI:** 10.1038/s41598-018-22549-6

**Published:** 2018-03-09

**Authors:** Michael Hornsey, David W. Wareham

**Affiliations:** 1 Antimicrobial Research Group, Centre for Immunology and Infectious Disease, Blizard Institute, Barts & The London School of Medicine and Dentistry, Queen Mary University London, London, UK; 2 Division of Infection, Barts & The London NHS Trust, London, UK

## Abstract

Multidrug-resistant lineages of *Acinetobacter baumannii* (MDRAB) are important nosocomial pathogens. As tigecycline remains active against most MDRAB we sought to investigate whether tigecycline resistance impacts biological fitness. The effects of treatment-emergent tigecycline resistance were investigated *in vitro* and *in vivo* using two pre- (AB210; W6976) and post-therapy (AB211; W7282) clinical pairs, recovered from individual patients, where tigecycline resistance was associated with up-regulated efflux activity. All isolates belonged to the same epidemic UK lineage. Significant differences were observed in end-point survival proportions between AB210 and AB211, but not between W6976 and W7282, using the *Galleria mellonella* infection model. Isolate AB211 outcompeted AB210 *in vivo*, in contrast to isolate W7282, which was outcompeted by its pre-therapy counterpart, W6972. Whole-genome sequencing of isolates W6976 and W7282 revealed a mutation in the *adeABC* regulatory gene, *adeS* in W7282; resulting in a Ser-8 → Arg substitution. Previous whole-genome comparison of AB210 and AB211 also identified a non-synonymous mutation in *adeS*, among several other lesions in genes involved in biofilm formation and DNA mismatch repair; consistent with the phenotypic differences described here. In conclusion, the differing effects on the wider phenotype were not predictable from the antibiograms or clonal lineage, despite a common mechanism of tigecycline resistance.

## Introduction

*Acinetobacter baumannii* is a Gram-negative opportunistic pathogen that has emerged in the last decade as one of the most problematic causes of healthcare-associated infection^1,2^. Once established the organism is extremely difficult to eradicate from the environment; it is capable of withstanding desiccation and the action of many disinfectants. Most strains also exhibit multidrug resistance (MDR), with many of the major epidemic clones that have disseminated worldwide retaining susceptibility to only polymyxins and tigecycline (TGC)^3,4^. Resistance to even these agents has now been described and in the case of TGC can occur due to up-regulation of efflux pumps of the resistance-nodulation-division (RND) family^5,6^, among other mechanisms.

Although a vast amount is known about mechanisms of antimicrobial resistance there is relatively little information on many of the basic processes contributing to the pathogenicity of *A. baumannii*. Amongst the processes that *A. baumannii* may use to establish human infection, studies performed *in vitro* suggest that it can readily forms biofilms, is able to adhere to and invade host cells^7,8^ and modulates the host immune response through an interaction with toll-like receptors (TLR) 2 and 4^9,10^. In terms of its ability to thrive within the human host many strains exhibit serum resistance^11^ and produce siderophores capable of scavenging iron from host proteins^12,13^. Other potential virulence factors include lipopolysaccharide^14,15^ production of exopolysaccharide and a capsule^16^.

The seemingly endless capacity of *A. baumannii* to develop resistance raises the question of whether this is costly to the organism. Knowledge of the biological cost of a MDR phenotype is important to gain a fuller understanding of the threat such isolates pose to human health.

The availability of susceptible and resistant pairs of clinical isolates obtained from the same patient offers an opportunity to study links between acquired resistance and virulence. These isolates have developed resistance over the course of a human infection, exposed to both the host immune system and antimicrobial chemotherapy. Previously we reported the *in vivo* emergence of TGC resistance in two MDR *A. baumannii* (MDRAB) isolates obtained from separate patients, in both cases in association with up-regulation of the AdeABC efflux system (Table 1)^5^. Data mining the whole-genome sequences of one of the pre-therapy (AB210; TGC-susceptible) and post-therapy (AB211; TGC-resistant) pair suggested that efflux-mediated TGC resistance might be associated with significant phenotypic differences^14^. In this study we assessed the impact of *in vivo* TGC exposure on the relative fitness and pathogenic potential of these MDRAB isolates using a range of *in vitro* and *in vivo* assays.

**Table 1:** Characteristics of *A. baumannii* isolates used.

Isolate	Origin	PFGE-assigned clone	MIC (mg/L)							
			AMP	CTX	IM	MEM	TOB	AMK	COL	**TGC**
AB210	Pre-therapy; clinical	OXA-23 clone 1	>64	>256	>32	>32	>32	>64	≤0.5	**0.5**
AB211	Post-therapy; clinical	OXA-23 clone 1	>64	>256	>32	>32	2	4	≤0.5	**16**
W6976	Pre-therapy; clinical	OXA-23 clone 1	>64	>256	>32	>32	>32	>64	1	**0.5**
W7282	Post-therapy; clinical	OXA-23 clone 1	>64	256	32	32	>32	>64	1	**8**

## Results

**Figure 1:**
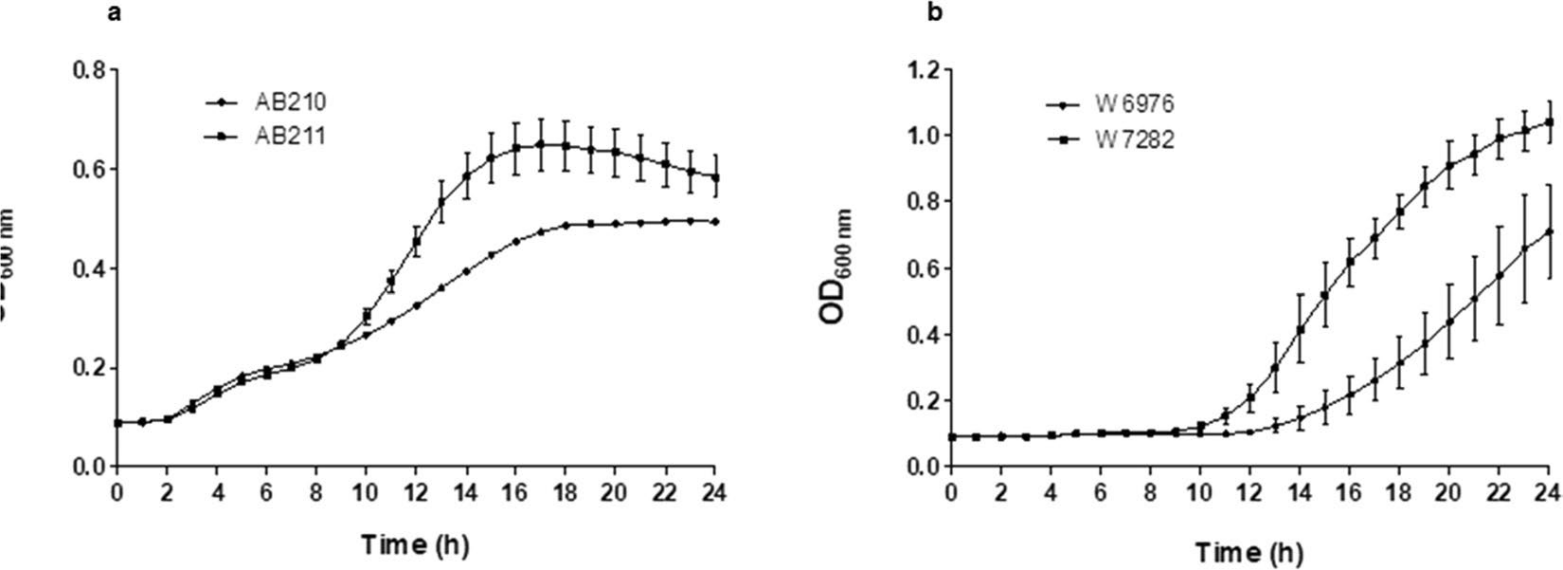
Growth curves of pre-and post-therapy isolates: (**a**) AB210 and AB211 in LB broth supplemented with 200 µM of the iron chelating agent, 2,2′-dipyridyl; (**b**) W6976 and W7282 in LB broth pH 4.5. Experiments were performed in triplicate. Error bars represent standard deviation.

Experiments conducted to compare the *in vitro* growth rates of each isolate revealed differences in their ability to grow under standard and ‘stressed’ laboratory conditions. Under most conditions, AB210 performed better in numerical terms than AB211, its TGC-resistant counterpart, but not to a statistically significant level. The exception was under iron limitation when AB211 was able to grow faster and to a higher optical density than AB210 in LB broth supplemented with 200 µM 2.2′ dipyridyl (Fig. 1a). Reproducible differences were also observed in the growth rates of the W6976 and W7282 pair. The post-therapy, TGC-resistant isolate was better able to grow at low pH (pH 4.5) (Fig. 1b) suggesting it might be better adapted for survival in acidic environments or acidic compartments of the host.

The ability of isolates to grow in the presence of bile was also assessed. All four organisms were bile-tolerant (growth in the presence of ≥10% bovine bile) although AB210 and AB211 were inhibited at a concentration of 16% w/v while W6976 and W7282 were not.

**Figure 2:**
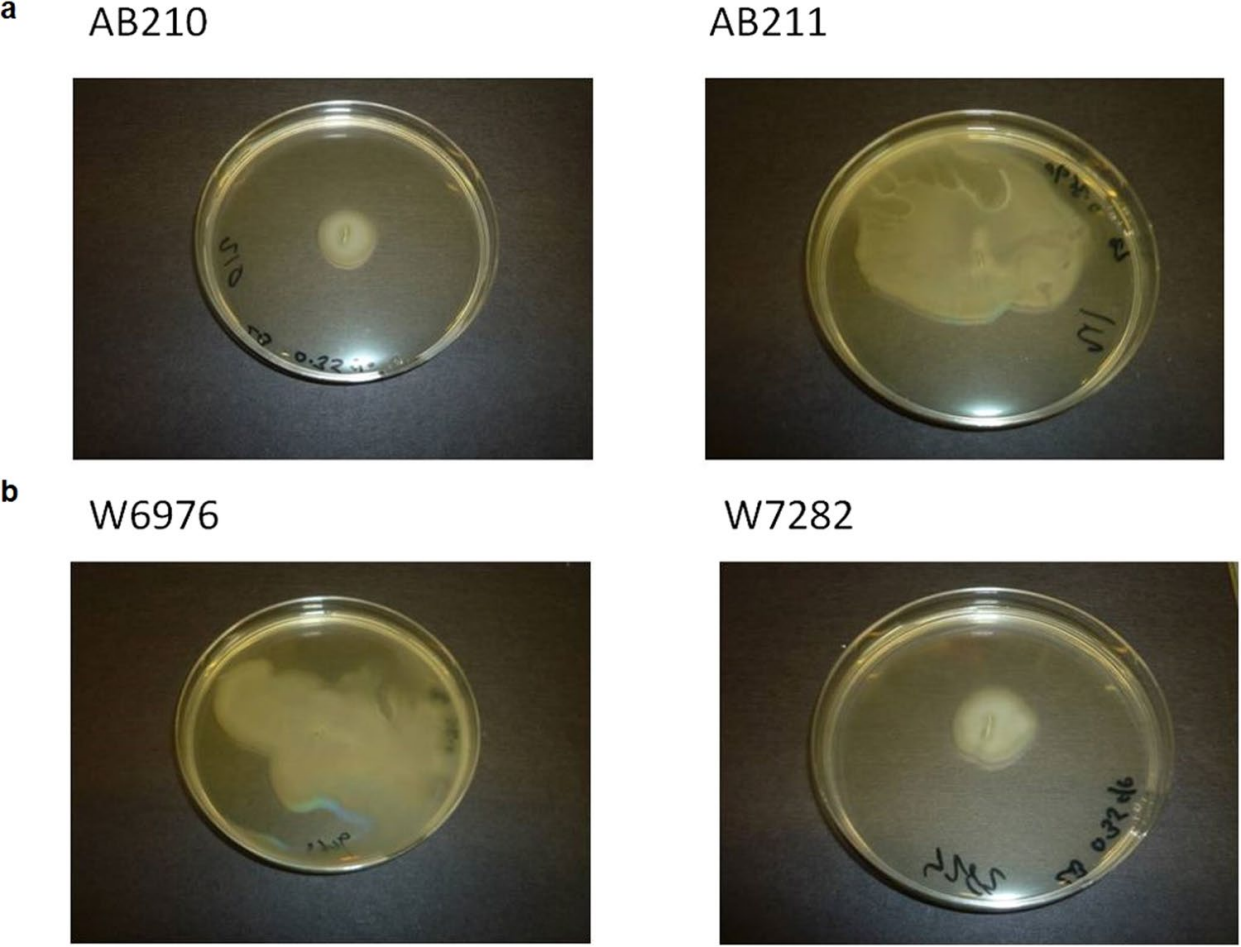
Results of the surface motility assay performed on low percentage (0.35%) LB agar plates: (**a**) AB210 (left) and AB211 (right); (**b**) W6976 (left) and W7282 (right).

**Figure 3:**
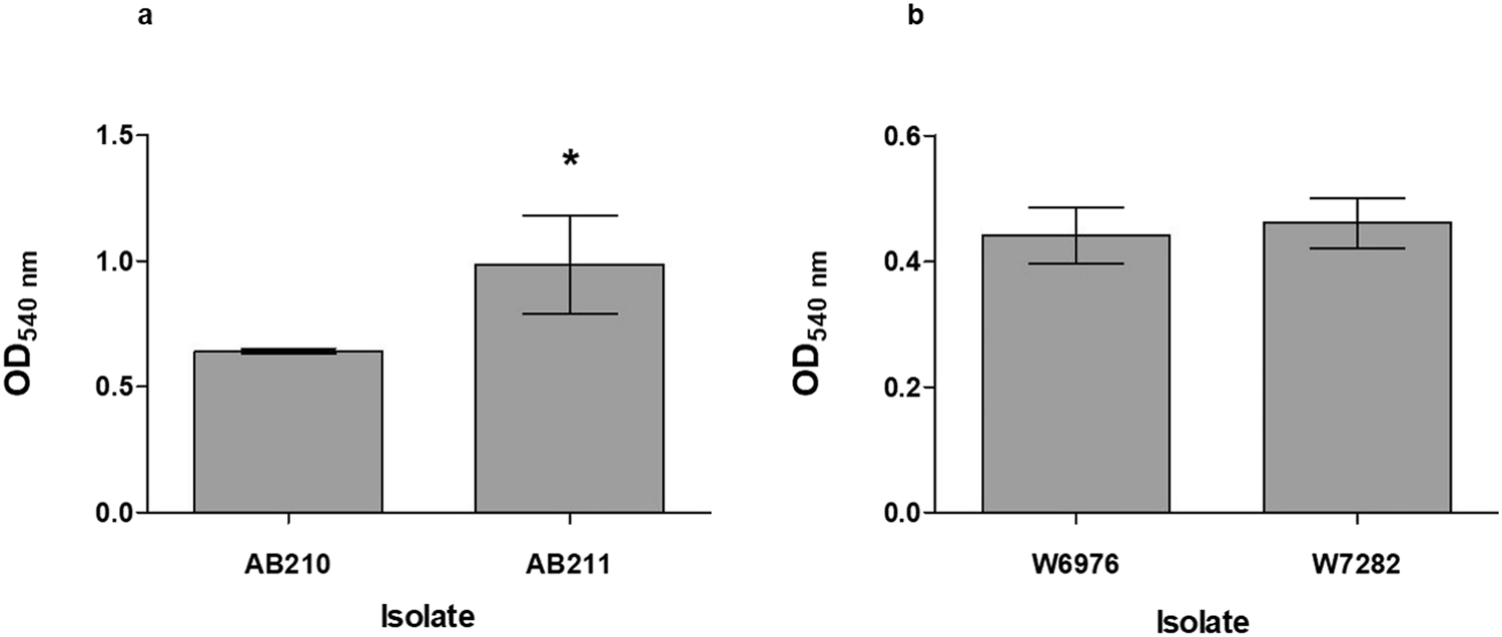
Biofilm production in the pre-and post-therapy isolates: (**a**) AB210 and AB211; (**b**) W6976 and W7282, assessed using a microtitre plate-based crystal violet assay. Experiments were performed in triplicate. Error bars represent standard deviation.

Pre-therapy isolate W6976 appeared to be motile whereas its post-therapy counterpart, W7282, was not. The opposite phenotypes were observed for AB210/AB211 (Fig. 2). When adherence to the wells of polystyrene microtitre plates was compared using a crystal violet assay, the motile, post-therapy isolate AB211 was better able to form a biofilm than AB210 (*p* = 0.0377), but no differences were found between W6976 and W7282 (*p* = 0.6024) (Fig. 3).

**Figure 4:**
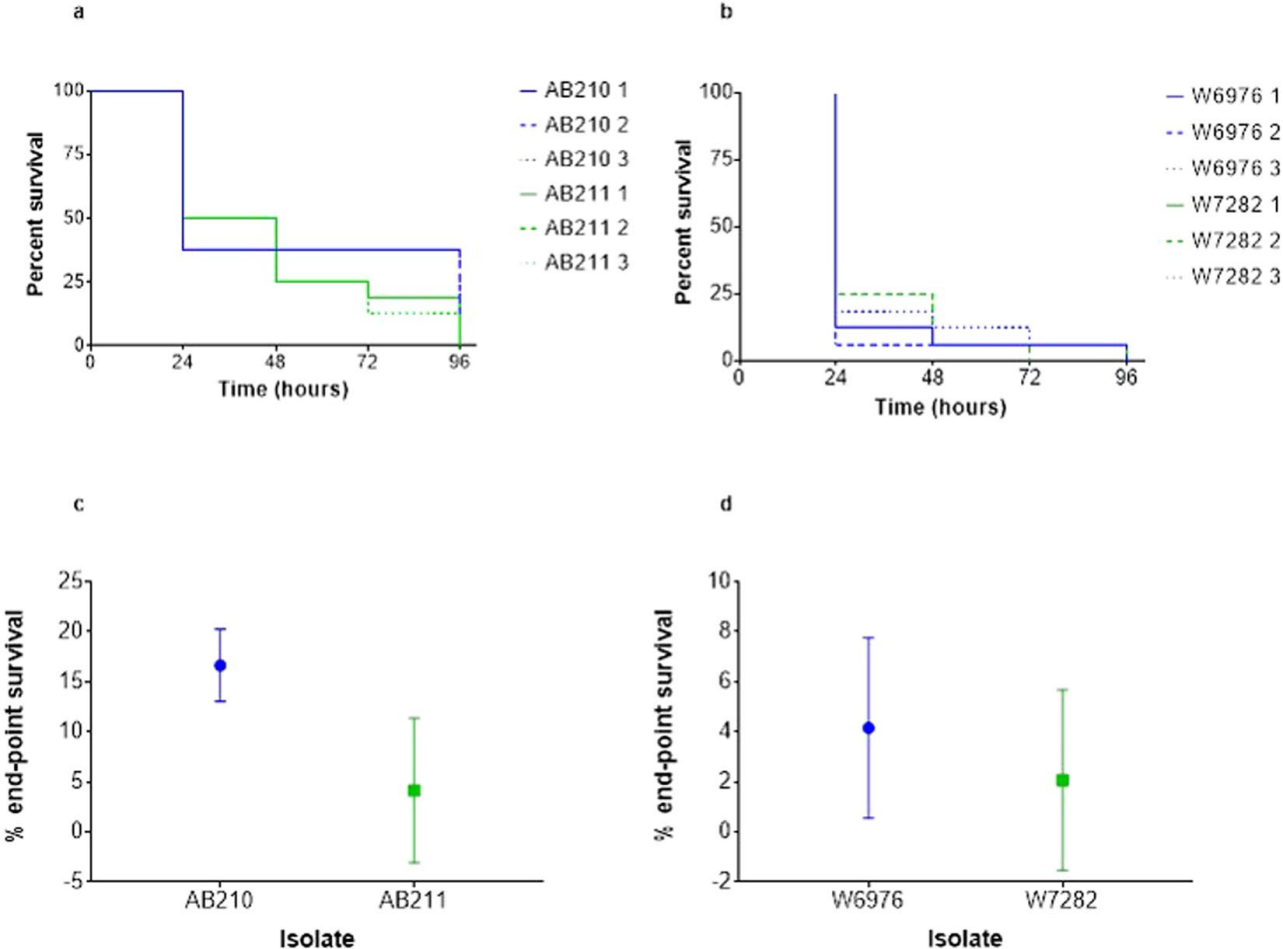
Survival curves and end-point survival proportions displaying *G. mellonella* killing: (**a**) AB210 (blue) and AB211 (green); (**b**) W6976 (blue) and W7282 (green); (**c**) average end-point survival percentages (AB210 and AB211); (**d**) average end-point survival percentages (W6976 and W7282). Data obtained from three biological replicates. Error bars represent standard deviation.

**Figure 5:**
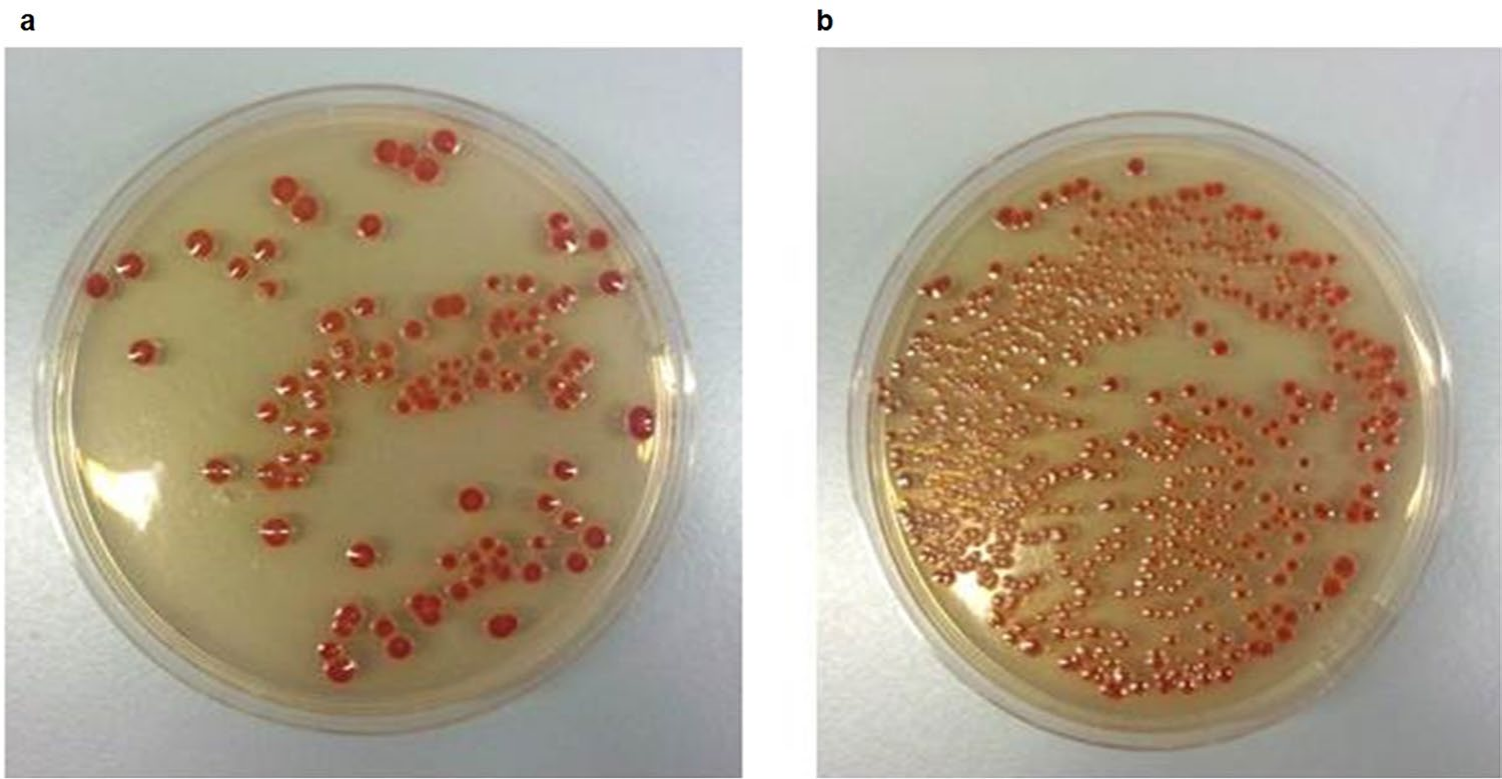
Images of plates from an *in vivo* competition assay (AB210 and AB211): (**a**) AB210 on CHROMagar *Acinetobacter* with KPC supplement containing 25 mg/L vancomycin and 25 mg/L kanamycin; (**b**) mixed population of AB210 and AB211 on CHROMagar *Acinetobacter* with KPC supplement containing 25 mg/L vancomycin.

Initial experiments demonstrated that all four *A. baumannii* isolates were pathogenic to *G. mellonella*. Analysis of survival curves revealed no reproducible differences in the killing kinetics of AB210 compared with AB211 or between W6976 and W7282 (Fig. 4a and b). However, analysis of end-point survival proportions indicated that AB211 was more virulent than AB210 (*p* = 0.0382), which was not the case for W7282 when compared with W6976 (*p* = 0.2593) (Fig. 4c and d). Growth of the isolates *in vivo* when in direct competition in the same animal was also investigated. After 24 hours incubation at 37 °C, post-therapy isolate AB211 was recovered from infected insects at a median 5-fold higher CFU/larvae compared with pre-therapy isolate AB210, suggesting that it could outcompete its TGC-susceptible counterpart *in vivo*. In contrast, pre-therapy isolate W6976 out-competed post-therapy isolate W7282 by a median ratio of 3:1 (Fig. 5).

Hypermutation has been shown to promote both the development of antimicrobial resistance, attenuated virulence, colonization and bacterial persistence in chronic infections^15,17^. AB211 was found to be a hypermutator as evidenced by the presence of significantly more colonies within the zone of inhibition around a fosfomycin disc compared with AB210 (*p* = 0.05). No such differences were observed between isolates W6976 and W7282 (*p* = 0.33) (Table 2).

**Table 2:** Hypermutator phenotype screening assay using 200 μg fosfomycin discs.

Isolate	Number of colonies within zone of inhibition			Median	Mann-Whitney *p* value (one-tailed)
	1st	2nd	3rd		
AB210	4	0	3	3	**0.05**
AB211	23	24	27	24	
W6976	5	5	4	5	**0.33**
W7282	6	0	0	0	

Whole-genome sequencing of W6976 (BioSample accession: SAMN02471603) and W7282 (BioSample accession: SAMN02471607) produced >40 million and >66 million nucleotide bases with a peak depth of eight- and 14-fold coverage, respectively, which gave estimated genome sizes of 3.91 and 3.95 Mb. Automated gene prediction of isolate W7282 detected 3825 putative coding sequences (CDSs), of which 3478 (91%) were homologous to the *A. baumannii* ACICU reference sequence (GenBank accession: NC_010611)^18^. Over 98% of the reads from W6976 mapped to the assembled W7282 genome (GenBank assembly accession: GCA_000248275.2).

Five putative SNPs were detected between the pre- and post- therapy isolates W6976 and W7282, although only one was non-synonymous. This SNP was located in the sensor histidine kinase gene, *adeS* and was predicted to result in a Ser-8 → Arg substitution. This mutation was not detected by Sanger sequencing when the nucleotide sequence of *adeRS* was originally investigated^5^. A larger number of SNPs (n = 18) were found between AB210 and AB211, 8 of which were non-synonymous. Of note, two mutations in AB211 (one nonsense; one non-synonymous) were found in genes encoding putative GGDEF domain containing proteins^14^. These molecules are thought to influence adhesion and biofilm formation via the second messenger cyclic-di-GMP, which regulates the shift from sessile to planktonic states^19^. The presence of these mutations in AB211 appears consistent with its motile phenotype and enhanced ability to form a biofilm compared with AB210. A deletion of the N-terminal region of *mutS*, a gene involved in DNA mismatch repair, was also found in AB211^14^.

## Discussion

The most pressing problem with MDR bacteria is a lack of drugs for effective treatment, though they also pose challenges for those involved in controlling their spread and quantifying the wider implications for public health. TGC and polymyxins are increasingly being used as treatments of last resort, especially for *A. baumannii* infections. With the threat of resistance to these agents now on the horizon it is timely to investigate how this might impact on the pathogenic potential of MDRAB.

Resistance is clearly advantageous to the organism in the presence of the drug, but the longer-term consequences and biological costs of maintaining a resistant phenotype in the absence of the selecting agent are less clear. Development of resistance via the acquisition of chromosomal mutations has often been associated with significant fitness costs *in vitro*^20–22^, but there are fewer studies that have examined this *in vivo*^23^. The effects of MDR due to the acquisition of additional genetic material via plasmids or transposons may be even more varied and will depend on whether virulence determinants are linked to the resistance genes^24^ and if there are metabolic costs associated with the maintenance of large MDR plasmids^25,26^.

We investigated the biological cost of TGC resistance selected for *in vivo* during on-label use of the drug for the treatment of MDRAB infections. In each case TGC resistance was likely due to up-regulation of the AdeABC efflux system. Links between antimicrobial resistance, RND pump activity and virulence have been reported for several organisms (e.g. *Escherichia coli*; *Salmonella enterica*^27^; *Pseudomonas aeruginosa*). Recently, several interesting studies have investigated the complexities of RND efflux pump regulatory systems and the associated impact on antimicrobial susceptibility and virulence in *A. baumannii*, both *in vitro* and *in vivo*^28–30^. However, to our knowledge this is one of the first studies to investigate these phenomena in *A. baumannii* using *in vivo*-selected, tigecycline susceptible and resistant clinical pairs recovered from individual patients.

*A. baumannii* can be found in the gastrointestinal tract of infected patients suggesting that gut colonisation may be important in the establishment and progression of infection^31^. In enteric bacteria such as *E. coli* and *S. enterica* RND transporters are thought to contribute to the ability of these organisms to survive in the gut as they can protect from the antimicrobial effects of bile salts. All the *A. baumannii* isolates were bile-tolerant, with no differences between isolates despite the changes in the expression of *adeABC*.

There were substantial phenotypic differences between the isolates that could clearly impact upon their pathogenic potential. TGC-resistant isolate AB211 was slower growing than AB210 under most laboratory conditions except under iron limitation. An enhanced ability to sequester this essential micronutrient may impart a competitive advantage on AB211 over AB210 *in vivo* where bacteria often encounter iron-limited environments. This is further supported by the competition experiments in *G. mellonella* where AB211 was recovered at a median 5-fold higher ratio than AB210. The ability to out-compete its susceptible counterpart in the absence of TGC exposure *in vivo* could impact on the transmissibility of AB211 in the nosocomial setting in which patients are heavily colonised. Overall, AB211 appeared to display more of a ‘persister phenotype’ with a low metabolic potential and better ability to form biofilms, which could in turn make it harder to eradicate from the environment.

The W6976/W7282 pair behaved quite differently from AB210/AB211. In the *G. mellonella* competition assay the TGC-susceptible isolate, W6976 was more successful, out-competing the resistant isolate by a median factor of 3:1. However, resistant isolate W7282 was better able to grow under acidic conditions (pH 4.5); the mechanism for this and any relation to RND pump activity is unclear although this may make it better adapted for survival in acidic environments, which would include parts of the gastrointestinal tract and within macrophages.

The acquisition of a resistance mechanism able to influence the normal physiology of the bacterial membrane, either by modulating the transfer of molecules across it (efflux changes) or by reducing its permeability (porin lesions) might usually be considered disadvantageous to the organism. This is supported by a study that investigated the effects of *in vivo-*selected imipenem resistance on the virulence of *Enterobacter cloacae*^32^. This study examined isolates that were either fully susceptible, had reduced susceptibility to imipenem due to enhanced activity of the AcrAB-TolC RND pump (AcrB exhibits 50% homology to AdeB in *A. baumannii*) or frank resistance when the pump lesion was combined with porin loss. In contrast to our observations on the virulence of TGC-resistant MDRAB in *G. mellonella*, all the imipenem-resistant *E. cloacae* isolates exhibited reduced growth and virulence in a *Caenorhabditis elegans* model. Interestingly, isolates which displayed only reduced susceptibility to imipenem due to up-regulation of the RND pump were more virulent in this model. We chose not to use the *C. elegans* model as small quantities of ethanol must be added to the media for it to be used in the study of *A. baumannii* pathogenesis^33^, ethanol exposure has a number of pleotropic effects on *A. baumannii*, including overexpression of *adeB*, which would have complicated our analysis^34^.

Multidrug resistance in the *A. baumannii* isolates used in this study was not known to be associated with porin loss. However, the effects of this have been investigated in a pan-drug resistant *A. baumannii* isolate lacking the outer membrane proteins CarO and OprD, although any contribution of efflux systems was not investigated. This isolate was found to exhibit impaired growth *in vitro* and had reduced cytotoxicity towards respiratory epithelial cells^35^, suggesting a significant cost. Nevertheless, these properties seemed to have negligible effect on the ability of the strain to cause an outbreak of infections in critically-ill patients^36^.

Comparison of the genome sequences of all four isolates revealed insights into possible reasons for the differences observed between the two pairs. Firstly, a greater number of SNPs were found between AB210 and AB211 (n = 18) than between W6976 and W7282 (n = 5). Indeed, the only obvious functional difference between W6976 and W7282 was the mutation encoding the substitution in the AdeS protein predicted to result in up-regulation of AdeABC. Although AB211 also harboured a mutation in *adeS* there were in addition several large deletions in the genome of this isolate compared with its counterpart. Of interest was a deletion of a portion of the *mutS* gene in AB211, a gene involved in DNA mismatch repair^37^.

The role of MutS in *Acinetobacter* genome plasticity has until recently largely been studied in the *Acinetobacter baylii* isolate, ADP-1. However, a recent study used a derivative of AB210 (AB201M) to evolve tigecycline resistance in a continuous culture system. Intriguingly, the researchers found that almost all the successful lineages became hypermutators because of the interruption of *mutS* by a mobile element. Moreover, *adeS* was found to be the most mutated gene^30^. Similarly, the post-therapy isolate AB211 exhibited a ‘hypermutator’ phenotype as evidenced by its increased capacity to develop spontaneous resistance to fosfomycin. It is possible that the *mutS* lesion was in fact the initial event that facilitated the selection and maintenance of the *adeS* mutation identified previously in AB211. Non-functional MutS could also be the reason for the ability of AB211 to out-compete AB210 *in vivo*. Any *in vivo* fitness cost associated with dysregulated RND activity alone, as suggested by the competition studies between W6976 and W7282, could be negated by the adaptive advantages of the *mutS* mutation.

The loss of TGC for the treatment of MDRAB leaves only polymyxins as viable treatment options. The prevalence of frank resistance to polymyxins in Gram-negative organisms varies widely by geographical region though is most common in the Enterobacteriaceae and *A. baumannii*^38^. Of most concern is the recent emergence, evolution and dissemination of plasmid-mediated phosphoethanolamine transferases in recent years. In a recent study, the introduction of the *mcr-1* gene into both laboratory and clinical strains of ESKAPE (*Enterococcus faecium*, *Staphylococcus aureus*, *K. pneumoniae*, *A. baumannii*, *P. aeruginosa*, and *Enterobacter* species) pathogens resulted in colistin resistance in *A. baumannii* among other species and consistent phosphoethanolamine modification of lipid A even in cases where only moderate increases in colistin MICs were observed^39^. However, to date there have been no reports of *A. baumannii* clinical isolates harbouring *mcr* genes. Literature evidence, along with the data presented here, highlight the need for a better understanding of the potential risks to public health posed by MDRAB beyond the antibiogram and clonal lineage.

## Methods

### Identification and characterisation of *A. baumannii* isolates

AB210, AB211, W6976 and W7282 were recovered from clinical samples using standard microbiology procedures and were partially characterised previously^5,14^ (Table 1).

### Comparative growth *in vitro*

*In vitro* growth rates were determined in LB broth using a microtitre plate-based growth kinetics assay^24^. Five microlitres of a 0.5 McFarland suspension were inoculated into 145 µL of growth medium in microtitre plates (Cornig, Amsterdam, Netherlands), which were incubated in a VersaMax Microplate Reader (Molecular Devices, Sunnyvale, CA, US) for 24 hours at 37 °C. The OD_600_ of the cultures was measured every 15 minutes with plates shaken for 10 seconds before each measurement. Growth under physiological stress (low pH, high salt concentration, nutrient and iron depletion) was investigated by growing the isolates in: (i) LB pH 4.5; (ii) LB supplemented with 200 mM NaCl; (iii) in one-third strength LB diluted with saline; and (iv) LB supplemented with 200 µM 2,2′-dipyridyl (an iron chelator); (v) normal, full strength LB. Relative fitness was calculated as *T*_*d*_ parent/*T*_*d*_ derivative. Differences in mean relative fitness were assessed using *t* test statistics.

### Bile tolerance assays

Sensitivity to bovine bile (Sigma-Aldrich, Poole, United Kingdom) was assessed *in vitro* using a microtitre plate-based assay. Fifty microlitres of 0.5 McFarland suspensions prepared in LB broth from overnight cultures were added to 150 μL of LB broth ± varying concentrations of bile (0–16% [w/v]). Plates were incubated at 37 °C without shaking overnight and examined for turbidity as a measure of microbial growth. Assay endpoints were confirmed by the addition of 20 µl of alamarBlue reagent (Life Technologies, Paisley, UK).

### Motility assays

Surface motility was assessed on low percentage agar plates. Five microlitres of overnight LB broth cultures were stab-inoculated into the centre of LB agar (0.3%) plates and incubated overnight at 37 °C. The ability of the organism to spread across the surface of the plate after overnight incubation was taken as evidence of motility^40^.

### Biofilm formation

Biofilm formation was assessed using a modified version of the methods described by King *et al*.^41^. Overnight LB broth cultures were used to inoculate fresh LB broths which were incubated at 37 °C with shaking until the OD_600_ reached 1.0. Fifty microlitres of these cultures were added to 50 µL LB broth in wells of a sterile, polystyrene microtitre plates and incubated overnight at 37 °C. After incubation, 100 µL of 1% crystal violet was added to the wells and the plate was incubated for 30 minutes at room temperature. Plates were washed three times using 200 µl of sterile distilled water, after which 200 µL of 95% ethanol was added to each well. One hundred and fifty microlitres of the ethanol from each well was transferred to a clean microtitre plate and the OD_540_ was measured.

### ***A. baumannii – Galleria mellonella*virulence assays**

The virulence of *A. baumannii* isolates was assessed by their ability to kill *G. mellonella* (wax moth) larvae using methods described by Peleg *et al*.^42^ Larvae were obtained from Livefood UK Limited (Rooks Bridge, Somerset, UK) and stored in the dark at 15 °C in wood shavings.

Briefly, an overnight LB broth culture of *A. baumannii* was washed twice in sterile saline and serially diluted. Using a 25 µL Hamilton syringe (Cole-Parmer, London, UK), 16 caterpillars were injected via a left proleg with 10 µL of diluted culture containing 10^4^ CFU. Un-inoculated and mock-inoculated (injected with 10 µL sterile saline) caterpillars were used as controls. Larvae were incubated in petri dishes lined with filter paper at 37 °C for 96 hours and scored daily for survival. Those insects that did not respond to touch with a pipette tip were considered dead. Experiments were performed on three separate occasions employing different batches of larvae. Survival curves were produced and analysed using the log-rank test. End-point survival proportions were compared using the one-tailed unpaired *t* test with Welch’s correction.

### ***In vivo*competition assay**

A selective culture medium, CHROMagar *Acinetobacter* (CHROMagar, Paris, France)^43^, was used to recover *A. baumannii* from infected *G. mellonella* larvae used in competition assays. This medium was supplemented with KPC supplement and vancomycin (25 mg/L) to prevent the growth of carbapenem-susceptible Gram-negative bacteria^44^ and Gram-positive bacteria, respectively. The ability of this media (CAA-KPC) to suppress growth of normal caterpillar flora was confirmed using un-infected larvae. Caterpillars were mechanically disrupted in 1 mL sterile PBS and 100 μL of the resulting suspension were plated on to supplemented CHROMagar *Acinetobacter* plates and ISO agar plates. For the recovery and enumeration of *A. baumannii* from larvae infected with AB211 or W7282 the CAA-KPC media was further supplemented with either kanamycin or TGC as detailed below.

The *in vivo* competition assay was performed using a starting ratio of 1:1 (AB210: AB211 or W6976: W7282) as determined by viable counts on ISO agar. After 24 hours incubation at 37 °C, the caterpillars were disrupted in 1 mL sterile PBS and serial dilutions were plated on CAA-KPC agar with and without either 25 mg/L kanamycin or 2 mg/L TGC. Ratios were calculated from viable counts on the selective media after 24 hours incubation at 37 °C.

### Hypermutation studies

All *A. baumannii* isolates were screened for a mutator phenotype using a modified version of the fosfomycin disc test method^45^. Briefly, 10^4^ CFU/ml from an overnight ISO broth culture was used to inoculate ISO agar plates. A disc containing 200 μg fosfomycin was placed on to the plates, which were then incubated for 24 hours at 37 °C. The numbers of colonies growing within the zone of inhibition around the fosfomycin disc were then counted. Differences in median number of colonies were assessed using Mann-Whitney statistics.

### Whole-genome DNA sequencing of isolates W6976 and W7282

Isolates W6976 and W7282 were grown on nutrient agar and genomic DNA sequenced using a 454 GS FLX pyrosequencer (Roche, Branford, CT, USA). Draft genomes were assembled *de novo* from flowgram data using Newbler v. 2.5 (Roche). The resulting contigs were annotated using the automated annotation pipeline on the xBASE server^46^. Single nucleotide polymorphisms (SNPs) detected in W6976 relative to W7282 were confirmed by PCR and Sanger sequencing of the resulting amplicons using an ABI 3730*xl* DNA analyser (Applied Biosystems, Warrington, UK).
